# Liver Lipids of Patients with Hepatitis B and C and Associated Hepatocellular Carcinoma

**DOI:** 10.3390/ijms22105297

**Published:** 2021-05-18

**Authors:** Elisabeth M. Haberl, Thomas S. Weiss, Georg Peschel, Kilian Weigand, Nikolai Köhler, Josch K. Pauling, Jürgen J. Wenzel, Marcus Höring, Sabrina Krautbauer, Gerhard Liebisch, Christa Buechler

**Affiliations:** 1 Department of Internal Medicine I, Regensburg University Hospital, 93053 Regensburg, Germany; haberl.elisabeth@gmx.de (E.M.H.); georg.peschel@klinik.uni-regensburg.de (G.P.); kilian.weigand@klinik.uni-regensburg.de (K.W.); 2 Children’s University Hospital (KUNO), Regensburg University Hospital, 93053 Regensburg, Germany; thomas.weiss@klinik.uni-regensburg.de; 3 LipiTUM Group, TUM School of Life Sciences Weihenstephan, Technical University of Munich, 85354 Freising, Germany; nikolai.koehler@tum.de (N.K.); josch.pauling@wzw.tum.de (J.K.P.); 4 Institute of Clinical Microbiology and Hygiene, Regensburg University Hospital, 93053 Regensburg, Germany; juergen.wenzel@klinik.uni-regensburg.de; 5 Institute of Clinical Chemistry and Laboratory Medicine, Regensburg University Hospital, 93053 Regensburg, Germany; marcus.hoering@klinik.uni-regensburg.de (M.H.); sabrina.krautbauer@klinik.uni-regensburg.de (S.K.); gerhard.liebisch@klinik.uni-regensburg.de (G.L.)

**Keywords:** polyunsaturated phospholipids, ceramide, triacylglycerol, p53

## Abstract

Hepatocellular carcinoma (HCC) still remains a difficult to cure malignancy. In recent years, the focus has shifted to lipid metabolism for the treatment of HCC. Very little is known about hepatitis B virus (HBV) and C virus (HCV)-related hepatic lipid disturbances in non-malignant and cancer tissues. The present study showed that triacylglycerol and cholesterol concentrations were similar in tumor adjacent HBV and HCV liver, and were not induced in the HCC tissues. Higher levels of free cholesterol, polyunsaturated phospholipids and diacylglycerol species were noted in non-tumorous HBV compared to HCV liver. Moreover, polyunsaturated phospholipids and diacylglycerols, and ceramides declined in tumors of HBV infected patients. All of these lipids remained unchanged in HCV-related HCC. In HCV tumors, polyunsaturated phosphatidylinositol levels were even induced. There were no associations of these lipid classes in non-tumor tissues with hepatic inflammation and fibrosis scores. Moreover, these lipids did not correlate with tumor grade or T-stage in HCC tissues. Lipid reprogramming of the three analysed HBV/HCV related tumors mostly resembled HBV-HCC. Indeed, lipid composition of non-tumorous HCV tissue, HCV tumors, HBV tumors and HBV/HCV tumors was highly similar. The tumor suppressor protein p53 regulates lipid metabolism. The p53 and p53S392 protein levels were induced in the tumors of HBV, HCV and double infected patients, and this was significant in HBV infection. Negative correlation of tumor p53 protein with free cholesterol indicates a role of p53 in cholesterol metabolism. In summary, the current study suggests that therapeutic strategies to target lipid metabolism in chronic viral hepatitis and associated cancers have to consider disease etiology.

## 1. Introduction

Chronic infections with hepatitis B virus (HBV) and hepatitis C virus (HCV) are leading causes for the pathogenesis of hepatocellular carcinoma (HCC), and are responsible for 60% to 85% of worldwide HCC cases [1]. Patients with chronic viral infections have a high probability to develop liver cirrhosis which is a relevant risk factor for carcinogenesis regardless of disease etiology [1]. Accordingly, common molecular pathways contribute to HCC development in HBV- and HCV-infected patients [2]. In HBV/HCV co-infected patients HCC is more frequent [3], suggesting that functionally distinct signaling molecules are also involved in HBV- and HCV-associated tumorigenesis [2]. The liver is the central organ in lipid metabolism, and subsequently, systemic lipoproteins were low in patients with liver cirrhosis [4]. Lipoproteins are particles composed of multiple lipid classes and serum cholesterol and sphingolipid levels declined in parallel with liver function [4].

Lipids are widely believed to be more essential for HCV than HBV infection. In particular HCV patients infected with genotype 3 often develop liver steatosis. Chronic HCV infection is related to insulin resistance and liver steatosis whereas HBV may even protect the patients from metabolic diseases [5].

The hepatic low-density lipoprotein (LDL)-receptor is essential in determining serum cholesterol levels, and is increased in the liver of HCV-infected patients. This indicates, that low LDL levels in HCV are a direct effect of viral infection [6,7]. Another study suggested that HBV virus also induced the LDL-receptor in HepG2 cells and thereby caused hepatic cholesterol accumulation [8].

Very few studies have compared the serum lipidome of HBV- and HCV-infected patients. Grammatikos et al. demonstrated associations of specific serum sphingolipid species with the severity of liver fibrosis and response to antiviral therapy in HCV patients. Such correlations did not exist in HBV-infected patients [9]. Importantly, C16:0 and C18:0 dihydroceramide, and C16:0 and C24:0 ceramide were low, whereas C24:1 ceramide was high in HCV compared to HBV serum [9]. On the other hand C16:0 and C18:0 dihydroceramide, and C16:0 and C24:1 ceramide were induced in HBV patients whereas C18:0 species declined in comparison with healthy controls [10]. Several serum sphingolipids were related to liver injury in chronic HBV infection. Moreover, a close association of seven sphingolipids with liver cirrhosis was described in these patients [10]. In a separate cohort of HBV-infected patients higher levels of serum phosphatidylcholine species and reduced levels of sphingomyelin species were identified in comparison to non-infected controls [11]. Interestingly, higher phosphatidylcholine in HBV infected HepG2 cells contributed to virus replication [11]. Depletion of phosphatidylcholine was, therefore, suggested as a therapeutic approach in HBV [11]. Furthermore, molecules preventing sphingolipid synthesis were supposed to inhibit HCV replication [12]. Serum lipids are related to liver function and are further affected by viral infection. These confounding factors must be considered in studies attempting to define lipid biomarkers for liver disease in chronic hepatitis. Thus, serum lipid signatures specific to HBV or HCV have not been finally established [4,9,13]. Moreover, lipid profiling of HBV and HCV infected liver was not described in detail so far.

It is generally accepted that lipid metabolism has a central role in cancerogenesis. In HCC tissues ceramide levels were diminished and this protects tumor cells from apoptosis [4]. Strategies to increase tissue ceramide levels are, therefore, potential therapeutic approaches for HCC [4,14]. Enhanced lipogenesis in cancer cells is essential for cell proliferation. Subsequent decline of polyunsaturated fatty acids and increase of saturated fat reduces cellular oxidative stress [4]. Drugs that impair lipogenesis may, therefore, protect from tumor growth [15]. However, these therapies have to consider the significant differences in inter-tumor lipid metabolism [16]. Comprehensive lipid profiling studies of HCC tissues did not compare tumors of patients with different disease etiologies [17,18,19,20,21] though this may be relevant for the tumor lipidome. Thus, there is still a need to define disease etiology related liver lipid composition in non-tumor and tumor tissues [4].

Abnormal lipid metabolism contributes to chronic liver diseases and HCC. Aim of the present study was the detailed analysis of the tumor and non-tumor lipidome of HBV and HCV infected patients to identify possible associations with disease etiology and severity.

## 2. Results

### 2.1. Lipidomic Analysis of Tumor and Adjacent Tissues of Patients with Chronic HBV and HCV Infections

**Figure 1:**
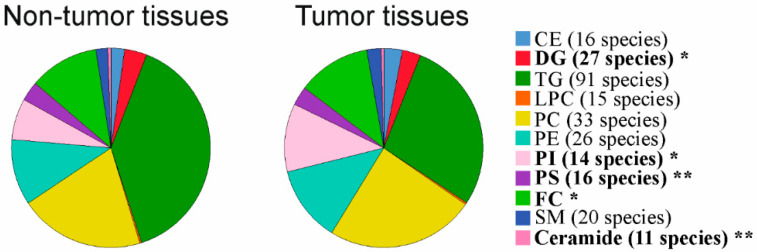
Lipidomic profiling in non-tumorous and tumor tissues of patients with chronic viral infection. Cholesteryl ester (CE), diacylglycerol (DG), triacylglycerol (TG), lysophosphatidylcholine (LPC), phosphatidylcholine (PC), phosphatidylethanolamine (PE), phosphatidylinositol (PI), phosphatidylserine (PS), free cholesterol (FC), sphingomyelin (SM) and ceramide levels in non-tumorous and tumor tissues of patients with chronic viral infection are shown. Number of species detected are given in brackets. Significant different lipid classes are in bold. * *p* < 0.05, ** *p* < 0.01 (Mann-Whitney U-test).

Lipidomic profiling was conducted in tumor and adjacent tissues of 10 HBV-, 11 HCV- and three HBV/HCV-infected patients. Triacylglycerols (TG) were one of the most abundant lipid classes and 91 different TG species could be detected. The second most common lipid class was phosphatidylcholine (PC) and 33 species could be specified. Phosphatidylethanolamine (PE, 26 species were determined) and free cholesterol (FC) were the third most common lipid classes and were equally abundant. This was followed by phosphatidylinositol (PI, 14 species), phosphatidylserine (PS, 16 species), cholesteryl ester (CE, 16 species), sphingomyelin (SM, 20 species) and diacylglycerol (DG, 27 species) levels. Ceramides (11 species) and lysophosphatidylcholine (LPC, 15 species) were the least common lipids in the liver (Figure 1). Lipidomic profiling of HCC tissues showed reduced levels of DG, PS, FC and ceramides, and increased PI levels in the tumors (Figure 1).

### 2.2. Triacylglycerols and Cholesterol in Non-Tumor Tissues of HBV and HCV Infected Patients

**Figure 2:**
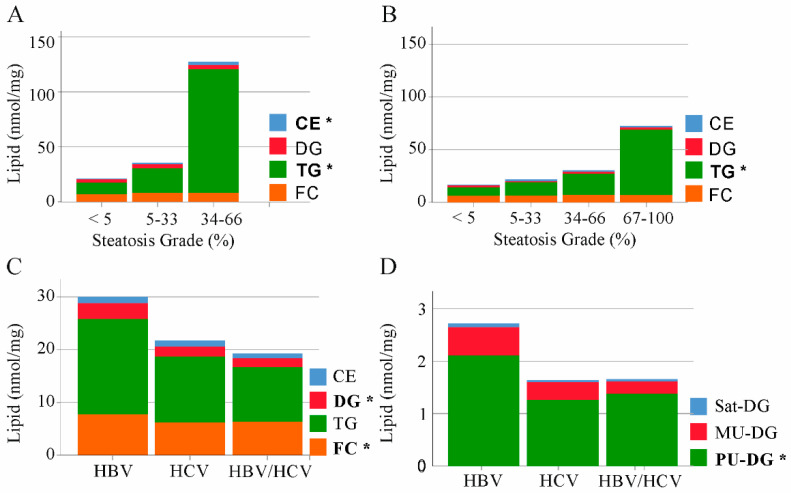
Cholesteryl ester (CE), diacylglycerol (DG), triacylglycerol (TG) and free cholesterol (FC) in non-tumor liver tissues of patients with HBV, HCV and HBV/HCV infection. (**A**) Median levels of CE, DG, TG and FC in HBV infected patients stratified for steatosis grade (<5%, seven patients; 5–33% one patient and 34–66% two patients). Lipids changed with increasing steatosis grade are in bold; (**B**) Median levels of CE, DG, TG and FC in HCV infected patients stratified for steatosis grade (<5%, six patients; 5–33% two patients, 34–66% one patient and 67–100% one patient; steatosis grade of one patients was unknown). Lipids changed with increasing steatosis grade are in bold; (**C**) Levels of CE, DG, TG and FC in non-tumor liver tissues of patients with HBV, HCV and HBV/HCV infection; (**D**) Saturated (Sat), monounsaturated (MU) and polyunsaturated (PU) DGs in non-tumor liver tissues of patients with HBV, HCV and HBV/HCV infection. Significant different lipid classes between HBV and HCV patients are in bold. * *p* < 0.05 (One-way ANOVA).

HCV replication uses the host’s lipid metabolism [5]. A common feature of chronic HCV infection is liver steatosis. [5]. Hepatic cholesterol accumulation via enhanced endogenous synthesis and upregulation of the LDL-receptor was described in HBV infection [8]. TGs, DGs and cholesterol are the major lipid classes, which accumulate in the steatotic liver [22]. Liver steatosis is graded according to the percentage of fat within the hepatocytes: grade 0 (<5%), grade 1 (5–33%), grade 2 (34–66%), and grade 3 (>66%) [23]. Hepatic CE and TG levels were positively associated with steatosis grade of HBV infected patients (Figure 2A). In the liver of HCV-infected patients TG levels were increased with higher steatosis grade (Figure 2B). FC and DGs were not changed with increasing steatosis grade in the fatty of liver of both groups (Figure 2A,B).

Comparison of TG and CE levels in the non-tumor liver tissues of HBV, HCV and double-infected patients revealed similar concentrations between the groups (Figure 2C). FC and DG levels were highest in the liver of HBV-infected patients, and these differences were significant in comparison to HCV patients (Figure 2C).

The degree of unsaturation affects the biological function of lipids and categorization in saturated, monounsaturated (MU) and polyunsaturated (PU) DG variants revealed that PU-DGs were high in HBV liver (Figure 2D).

HCV genotype 3 is associated with liver steatosis, and this is a direct effect of the virus [24]. Genotype of seven HCV infected patients was documented. Preliminary analysis could not identify higher levels of CE, DG, TG or FC in the two genotype 3 patients in comparison to the four genotype 1-infected patients (Supplementary Figure S1).

### 2.3. Phospholipids in Non-Tumor Tissues of HBV and HCV Infected Patients

**Figure 3:**
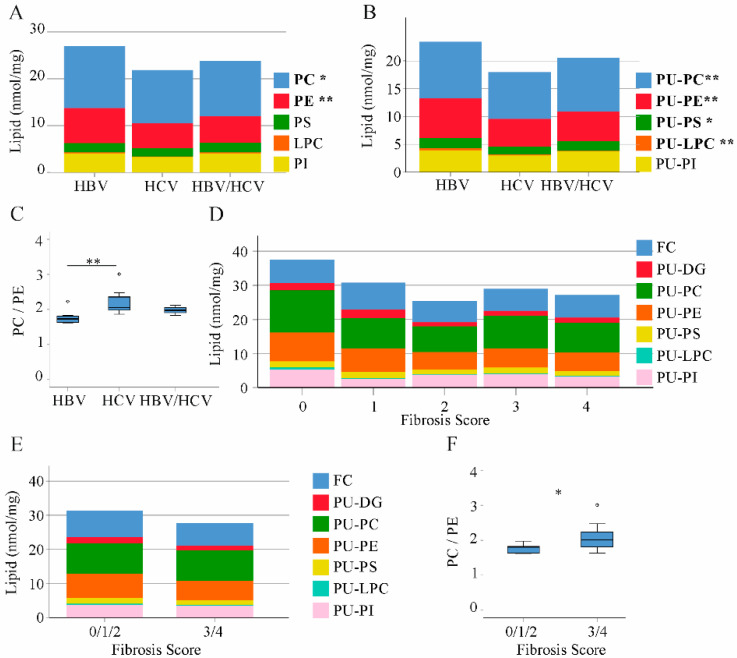
Phospholipid, diacylglycerol and free cholesterol levels in non-tumor liver tissues of patients with HBV, HCV and HBV/HCV infection. (**A**) Phosphatidylcholine (PC), phosphatidylethanolamine (PE), phosphatidylserine (PS), lysophosphatidylcholine (LPC) and phosphatidylinositol (PI) levels in the liver of HBV, HCV and double infected patients; (**B**) Polyunsaturated (PU) phospholipids; (**C**) PC/PE ratio; (**D**) free cholesterol (FC), PU-diacylglycerol (DG) and PU-phospholipids in patients with increasing fibrosis stages; (**E**) FC, PU-DG and PU-phospholipids in patients with fibrosis scores 0/1/2 and fibrosis scores 3/4; (**F**) PC/PE ratio in patients with fibrosis scores 0/1/2 and 3/4. * *p* < 0.05, ** *p* < 0.01 for comparison of HBV and HCV. (One-way ANOVA).

Phospholipids are highly abundant in the liver (Figure 1). PC and PE levels were higher in HBV than HCV liver. Total levels of PS, LPC and PI did not vary between the groups (Figure 3A). Whereas saturated and MU phospholipid variants were equally abundant in the liver of HBV and HCV infected patients, PU-PC, PU-PE, PU-PS and PU-LPC were higher in HBV than HCV-infected liver tissues (Figure 3B and Table 1).

The hepatic PC/PE ratio ranges between 1.5 and 2.0 in the healthy liver [25]. Median value was 1.7 in HBV, 2.1 in HCV and 2.0 in double infected patients illustrating abnormally increased PC/PE ratio in HCV (Figure 3C).

HCV-infected patients had a higher fibrosis grade (Table 2) and this may contribute to differences in PU-phospholipid and FC levels between HBV- and HCV-infected patients. FC, PU-DG, PU-PC, PU-PE, PU-PS, PU-LPC and PU-PI did not correlate with fibrosis score and did not consistently decline with higher fibrosis stage in the whole cohort and when calculated separately in both groups (Figure 3D and data not shown). When patients with 0, 1 and 2 fibrosis stages were combined in a group the PC/PE ratio was lower than in patients with fibrosis stage 3 or 4. Levels of PU-phospholipids were similar between these two groups (Figure 3E,F).

Altogether, these data showed major differences in hepatic phospholipid composition of HBV and HCV infected patients. HBV/HCV liver tissues had lipid levels more similar to HCV positive probands. Here, only three patients were included and further analysis has to confirm this preliminary finding.

### 2.4. Ceramide and Sphingomyelin in Non-Tumor and Tumor Tissues of HBV and HCV Infected Patients

**Figure 4:**
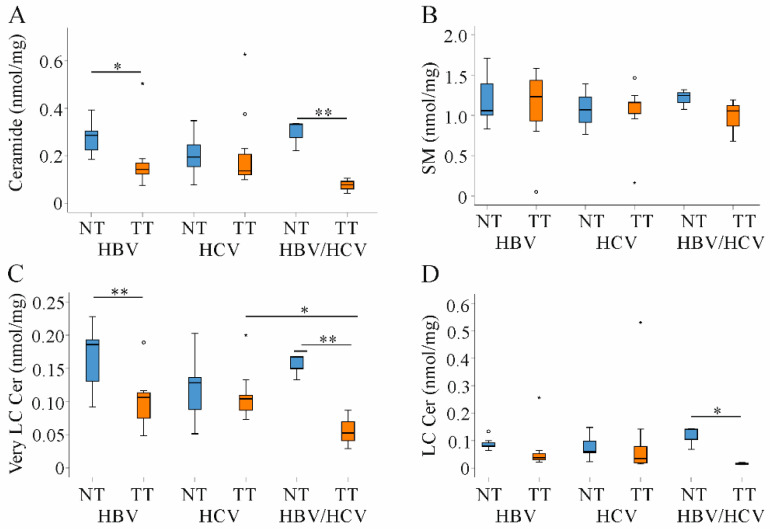
Ceramide (Cer) and sphingomyelin (SM) in non-tumor (NT, blue boxes) and tumor tissues (TT, orange boxes) of patients with HBV and HCV infection. (**A**) Ceramide; (**B**) SM; (**C**) Very long-chain ceramide (Very LC Cer) and (**D**) Long-chain ceramide (LC Cer) levels in NT and TT of HBV, HCV and double infected patients. Small circles and stars in the figures identify outliers. * *p* < 0.05, ** *p* < 0.01 (Kruskal-Wallis-Test).

Ceramide has a central role in cell death and inflammation, and was similar in the non-tumorous liver tissues of all groups. This also applied for sphingomyelin (SM) levels (Figure 4A,B).

In cancer tissues ceramide levels are usually suppressed. Low ceramide levels were described in non-viral and HBV HCC tissues [18,19,21]. In the patients studied here, ceramide declined in tumors of HBV and HBV/HCV-infected patients and was quite normal in HCV-related HCC tissues (Figure 4A). Though recent studies suggested impaired sphingomyelinase activity as the major cause of low ceramide concentrations [18,19,21], SM levels were not concordantly changed in the tumors (Figure 4B).

Ceramides in relation to the length of their acyl-chain have distinct biological functions. In fact, long-chain ceramides (C_16_–C_20_) induced apoptosis and oxidative stress whereas very long-chain ceramides (C_22_–C_24_) had opposite activities [4,26]. Very-long chain ceramides declined in tumors of HBV and HBV/HCV infected patients. Long-chain species tended to be lower in HBV and were significantly reduced in double infected patients (Figure 4C,D).

### 2.5. Cholesterol, Tri- and Diacylglycerides in Tumor Tissues of HBV and HCV Infected Patients

**Figure 5:**
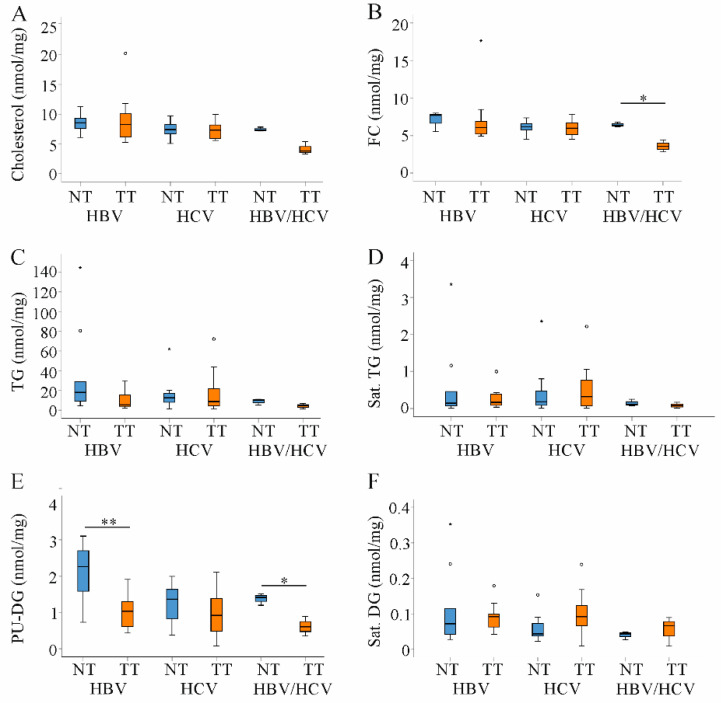
Cholesterol, triacylglycerols (TG) and diacylglcerols (DG) in non-tumor (NT, blue boxes) and tumor tissues (TT, orange boxes) of patients with HBV and HCV infection. (**A**) Cholesterol; (**B**) Free cholesterol (FC); (**C**) TG; (**D**) Saturated (sat.) TG; (**E**) Polyunsaturated (PU) DG and (**F**) Sat. DG in NT and TT of HBV, HCV and double infected patients. Small circles and stars in the figures identify outliers. * *p* < 0.05, ** *p* < 0.01 (Kruskal-Wallis-Test).

Cholesterol is another lipid which contributes to tumor growth [4]. Total cholesterol, CE and FC levels were unchanged in HBV- and HCV-associated HCC. In HBV/HCV-infected patients FC was reduced in the tumors (Figure 5A,B and data not shown). TG species were found increased in HCC tissues of patients where most cases were related to HBV [19,21]. Surprisingly, TG levels were not changed in the tumors of any patient group (Figure 5C). Moreover, there was no shift from PU to saturated TG species (Figure 5D and data not shown). DGs are precursors for TGs, and PU derivatives declined in tumors of HBV and double infected patients and were unchanged in HCV (Figure 5E). Saturated DG levels did not change in the tumor tissues (Figure 5F).

### 2.6. Decreased PU-Phospholipids in HBV Tumors

Previous studies identified a decline of PU-PC, PU-PE and PU-PS in non-viral and HBV-HCC [18,19]. In good agreement with these findings PU-PC, PU-PE, and PU-PS declined in HCC tissues of HBV infected patients. Saturated PC was increased (Table 1). In HCV patients saturated PC and PU-PI were the only phospholipids changed in the tumors and were induced in HCC tissues. PC/PE ratio did not change in the tumors (Table 1). LPC levels remained unchanged in the tumors of all patients (Table 1).

### 2.7. Correlation of Lipids in HCC Tissues with Tumor Grading and Stage

**Figure 6:**
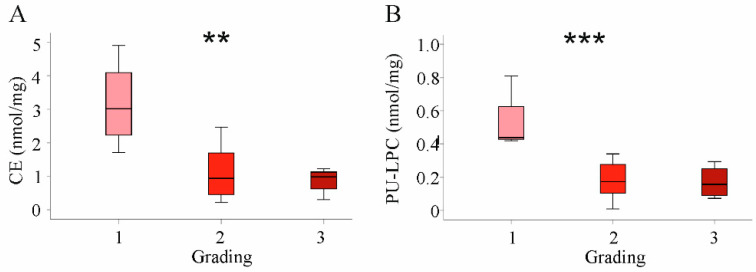
Association of cholesteryl ester (CE) and polyunsaturated lysophosphatidylcholine (PU-LPC) with tumor grading in the whole cohort. (**A**) CE levels and (**B**) PU-LPC levels in HCC tissues stratified for tumor grading. ** *p* < 0.01, *** *p* < 0.001 (One-way ANOVA).

All lipid classes described above had similar levels in HBV- and HCV-associated HCC. Therefore, correlation analysis with tumor grade and stage was performed in the whole cohort. Significant associations were, however, not identified (data not shown). Nevertheless, there was a significant decline of CE and PU-LPC levels with increasing tumor grade (Figure 6). Such associations did not exist for T-stage (data not shown). Subgroup analysis could not identify any associations between lipids, tumor grading and stage most likely because of the small number of patients per group.

### 2.8. Expression of p53 Protein in HBV and HCV Infected Patients

The tumor suppressor protein p53 controls various processes involved in cell proliferation and tumor growth. More recent studies showed that p53 regulates lipid metabolism [27].

**Figure 7:**
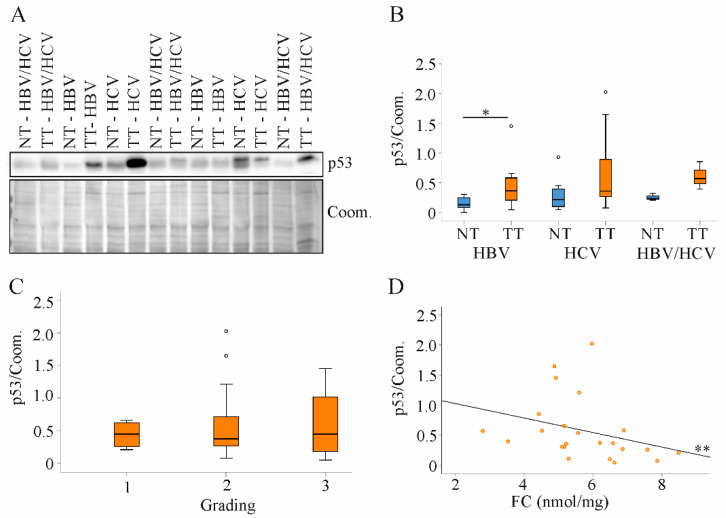
Expression of p53 protein in non-tumor tissues and tumor tissues of HBV, HCV and HBV/HCV patients. (**A**) Representative immunoblot of p53 protein analysis; (**B**) Quantification of p53 protein in non-tumor tissues (NT) and tumor tissues (TT) of all patients. Coomassie (Coom.) stained membrane was used as loading control; (**C**) Tumor p53 protein in relation to tumor grading; (**D**) Correlation of p53 protein and free cholesterol (FC) in tumor tissues of all patients. One patient in the HBV group and three patients in the HCV group had p53 protein levels greater than 1. * *p* < 0.05, ** *p* < 0.01 (Kruskal-Wallis-Test, One-Way ANOVA and Spearman).

The p53 protein levels were induced in the tumors, and this was significant for the HBV infected patients. HBV, HCV and HBV/HCV infected patients had comparable p53 protein in normal and tumor tissues (Figure 7A,B).

Tumor tissue p53 protein levels were not associated with grading or T-stage (Figure 7C and data not shown). There was a negative correlation of tumor p53 protein levels with FC (Figure 7D).

**Figure 8:**
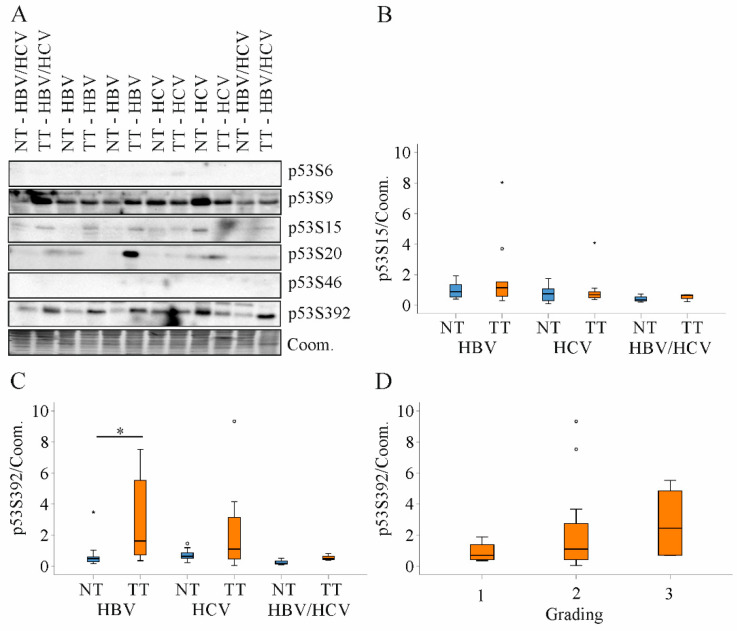
Expression of p53S6, S9, S15, S20, S46 and S392 protein in non-tumor tissues and tumor tissues of HBV, HCV and HBV/HCV patients. (**A**) Representative immunoblot of p53 protein analysis; (**B**) Quantification of p53S15 protein in non-tumor tissues (NT) and tumor tissues (TT) of all patients. Coomassie (Coom.) stained membrane was used as loading control; (**C**) Quantification of p53S392 protein in NT and TT of all patients. Coomassie (Coom.) stained membrane was used as loading control; (**D**) Tumor p53S392 protein in relation to tumor grading. * *p* < 0.05 (Kruskal-Wallis-Test, One-Way ANOVA).

The function of p53 is regulated by posttranslational modifications. Phosphorylation at serine (S) 6, 9, 15 or 20 blocks binding to mouse double minute 2 homolog (MDM2) and thereby increases p53 protein stability [28]. P53S6 was not detected by immunoblot, and p53S9, S15 and S20 were not changed in the HCC tissues (Figure 8A). Expression in non-tumor tissues of HBV and HCV patients was comparable (Figure 8A,B). Phosphorylation of p53 at S46 or at S392 promotes apoptosis [28]. Immunoblot analysis could not detect p53S46 (Figure 8A). P53S392 was induced in the tumors, and this was significant in HBV patients (Figure 8A,C). Tumor p53S9, S15, S20 or p53S392 levels were correlated with each other but did not correlate with p53 protein levels, grading, T-stage or FC (Figure 8D and Table 2).

## 3. Discussion

The current study confirmed the already described decline of ceramide and PU phospholipids in HCC tissues of HBV-infected patients [4]. PU phospholipids were already low in non-tumor tissues of HCV-infected patients, and a further decrease in the tumors was not observed. Ceramide levels did not decline in HCC tissues of HCV patients. Considering that PU phospholipids and ceramide decreased in tumors of patients with HBV, non-alcoholic steatohepatitis (NASH) and cryptogenic disease etiology [4], HCV infection is an exception in this regard.

It is well known that HCV infection upregulates the expression of the hepatic LDL-receptor and thereby enhances uptake of LDL in hepatocytes [7]. LDL-receptor protein was in fact induced in the liver of HCV infected patients in comparison to non-HCV patients [6,7].

Hepatic cholesterol accumulation via enhanced endogenous synthesis and upregulation of the LDL-receptor was also described in HBV infection [8]. LDL-receptor protein was indeed higher in HBV- and HCV-infected liver when compared to the liver of patients with non-viral liver diseases [6]. LDL particles have a high content of CE, PC, SM and LPC [13], and various LPC and SM species were induced in the liver of LDL-receptor knockout rats [29]. HBV and HCV liver had comparable levels of CE, SM and LPC and this is principally in line with a similar activity of the LDL-receptor uptake pathway.

HCV infection was shown to enhance hepatic lipogenesis in comparison to healthy controls [30]. Lipogenesis was also induced by HBV infection [31], and current analysis showed that levels of hepatic TGs were similar in HBV and HCV liver. HCV genotypes differ in their capacity to induce liver steatosis [32]. Genotypes were only documented for seven patients and this preliminary analysis could not identify differences in hepatic TG levels.

HCV infection strongly impairs whole body and hepatic cholesterol synthesis [30]. HCV eradication induces serum LDL suggesting that low concentrations of cholesterol are a direct effect of HCV infection [30,33]. HBV rather upregulates genes involved in cholesterol biosynthesis [34], and accordingly, FC levels, which are about 6 times higher than CE concentrations, were reduced in HCV liver.

Of note, histologically evaluated liver steatosis was linked to higher TG and CE levels in HBV infected patients. In HCV, only TG concentration increased with higher steatosis grade. HCV restricts hepatic cholesterol levels [30], and thus, cholesterol may not accumulate in the fatty liver.

PU lipids are low in patients with liver cirrhosis, and insufficient hepatic production, malnutrition, and reduced vitamin E levels were supposed as cause [35]. A separate analysis described that docosahexaenoic acid but not docosapentaenoic acid or arachidonic acid declined in the cirrhotic liver [36]. In the current study population, PU-phospholipids and PU-DG were not associated with fibrosis stage. Though the PC/PE ratio was induced in patients with advanced fibrosis in comparison to patients with milder disease stages, PU-lipid levels did not decrease in parallel.

PU-phospholipids were low in the membrane of red blood cells of HCV infected patients and supplementation with vitamin E increased the ratio of PU to saturated fatty acids [37]. Oxidative stress was, however, increased in chronic HBV and HCV infection [38], and this excludes oxidative stress as a major cause of PU-phospholipid depletion in HCV.

PC in the liver originates from the CDP-choline pathway, by the activity of phosphatidylethanolamine N-methyltransferase (PEMT) or is derived from plasma lipoproteins [25,39]. Lower PC levels were described in HCV-infected cells and were attributed to reduced choline uptake and PEMT expression [40,41]. The metabolic pathways of phospholipids are interrelated [25,39], and HCV infection induced decline of cellular PC concentration may also effect on further phospholipids [40]. Phospholipids can act as a source of DG generation [42], which were also reduced in HCV infected cells. In the liver PU phospholipids and PU DGs were the most abundant lipids, and all declined upon HCV infection.

Increased hepatic lipogenesis also contributes to PU-lipid depletion [4] and hepatic lipogenesis was enhanced in HCV patients in comparison to healthy controls [30]. TGs did, however, not accumulate in HCV liver in comparison to HBV infected patients. HCV blocks the hepatic release of TG-rich very low-density lipoprotein particles [43] and further impairs beta-oxidation of lipids [44] showing that these pathways will not compensate for increased de novo lipogenesis. Because TG levels were similar in all of the patients analyzed, higher lipogenesis in the liver of HCV patients is unlikely.

The current analysis also included three patients infected with both viruses. Even though most of the observed changes in lipid levels were not significant, it was obvious, that lipid composition of non-tumor tissues was more similar to HCV than HBV patients.

Abnormal lipid composition has a critical role in carcinogenesis and is a target for new HCC therapeutics [4]. Detailed analysis of lipids is fundamental for the identification of druggable pathways. Exogenous ceramide induced apoptosis of cancer cells, and seems to be also effective in sorafenib-resistant HCC [14]. Cellular ceramide was lowest in HCC of double-infected patients, and strategies raising its levels to induce apoptosis of tumor cells may be most effective in this cohort. Of note, very long chain and long chain ceramides were low in the HCC tissues of patients infected with HBV and patients infected with HBV/HCV.

Ceramide was shown before to decline in HCC tissues [4]. In these studies, tissues of HBV infected patients and patients with non-viral disease etiology were analyzed. Though these recent studies described increased SM in the paratumorous tissues [18,19,21] such an induction was not observed in the present analysis. The decline of ceramides in the tumors was not associated with higher SM levels excluding a major role of sphingomyelinases. Ceramide can be converted to sphingolipids such as glycosylceramide or sphingosine-1-phosphate [45] and future studies have to identify the pathways which contribute to low ceramide levels in HCC tissues. Of note, a decrease of ceramide levels was not observed in HCV patients.

Intake of dietary choline was inversely associated with HCC risk in HBV infected patients and strategies targeting phospholipid levels may be effective in HCC therapy of these patients [46]. Interestingly, levels of PU-PC, PU-PE and PU-PS were reduced in HCC tissues of HBV patients. In HCV patients these lipids were not altered in the tumors, and PU-PI was even induced. A shift from PU to saturated lipids protects the cells from oxidative stress and is critical for cell proliferation [4,47]. This does obviously not apply to cancerogenesis in HCV infected patients. Risk of HCC is not higher in HCV than HBV patients [48]. Moreover, PU-lipids were reduced in non-malignant HCV liver, but this does not predispose the patients to tumorigenesis [48]. Regarding that only two lipid classes (PU-PI and sat. PC) were changed in HCV tumors, lipid remodeling may be less important in cancerogenesis in these patients.

Increased lipogenesis in HCC tissues was described in previous studies, and it was suggested that blockage of this pathway may prevent liver cancer [15]. TG levels were not enhanced in HBV and HCV-related HCCs, and such strategies may not be efficient for HCC therapy of these patients.

Lysophosphatidylcholine acyltransferase (LPCAT) 1 is upregulated in tumor cells and generates saturated PCs from LPC [47]. Whether this pathway contributes to PU-PC depletion in HCC tissues or the decline of PU-LPC with increasing histological tumor grade needs further analysis. The negative association of CE with tumor grade may be an indicator for the high cholesterol demand of rapidly proliferating cells [49].

HCC-induced alterations in the double infected patients were closer to HBV patients. Of note, a strong decline of FC was observed in HCC tissues of those patients whereas levels were neither changed in the HCV nor HBV group. FC is cytotoxic and low levels could contribute to tumor growth [50]. HBV and HCV coinfections increase the risk for HCC [51], and present study suggests that the strong suppression of FC and ceramide levels may contribute to enhanced cell proliferation.

CEs were comparable in non-tumor and tumor tissues of all of the patients. Previous studies reported on high cholesterol in HCC tissues, and a further study described that CEs, but not FC, were induced [18,52]. Disease etiology was not specified in the first study, and was non-viral in the second one [18,52]. Thus, aberrant cholesterol in HCC may be related to disease etiology, and this needs further investigation. This is also the case for ceramides which declined in non-viral [4], HBV and HBV/HCV related tumors. In HCV patients tumor and non-tumor ceramide levels were equal.

There is growing evidence that the tumor suppressor protein p53 is involved in lipid metabolism [53]. Protein levels of p53 were similar in HBV and HCV liver suggesting a minor, if any role, of p53 in the variations of the hepatic lipid composition.

Mutated and wild type p53 can exert oncogenic functions [54,55]. Protein levels of p53 were induced in HCC tissues, and this was significant for HBV-infected patients. Upregulation of p53 protein in non-viral HCC was shown before, and mutated as well as wild type p53 were increased in the tumors [18,56]. Tumor p53 protein negatively correlated with FC levels. Overexpression of wild-type p53 protein decreased FC in fibroblasts [57], and a similar regulation may exist in hepatocytes.

Diverse mechanisms regulate p53 activity and phosphorylation of serine residues across the whole protein have been identified [28]. Phosphorylation of S46 is induced by DNA damage and enhances apoptosis. Accordingly, p53S46 was not detected in the liver tissues analyzed. Phosphorylation at S392, which may promote mitochondrial translocation of p53 and apoptosis [28], was nevertheless induced in the tumor tissues. Phosphorylation of p53 at S392 is induced by diverse stimuli, which stabilize p53 protein [58]. P53S6, S9, S15 and S20 protect p53 from Mdm2 binding, and enhance p53 stability and function [28]. P53S6 was not detected by immunoblot indicating low expression in the liver. Levels of the further isoforms did not differ between HBV and HCV and were not higher in the tumors. There are multiple other phosphorylation sites, and posttranslational modifications such as acetylation also control p53 function. The role of most of these modifications has not been finally resolved. It is likely that p53 activity is controlled by multiple modifications and not by phosphorylation of a single-site [28]. High p53S15 expression in HCC tissues of mostly HBV infected patients was related to longer survival [59]. Such an association did not exist for p53S392 [59]. Correlations between p53S15 and clinicopathologial factors such as vascular invasion were not identified in that study. Of note, p53S392 was positively associated with intrahepatic invasion [59]. Both of these isoforms positively correlated with p53 protein levels in the HCC tissues [59]. In the HCC tissues studied herein, phosphorylated isoforms correlated with each other but none of these isoforms was associated with p53 protein levels. Posttranslational modifications of p53 at one amino acid can affect modifications at different sites [28], but whether this applies for phosphorylation of p53 at different serine residues was not described to our knowledge so far.

A major limitation of the present study is that the number of patients, and especially of patients simultaneously infected with HBV and HCV virus, was rather small. It was nevertheless possible to confirm HCC associated lipid changes already described in previous studies [4]. Most investigations so far analyzed lipid signatures in serum which is more easily accessible than liver tissues [4]. Liver tissues of healthy probands were not analysed for ethical issues and tissues of patients with non-viral HCC were not included. The main etiologies of non-viral HCC are alcohol abuse and NASH [4,60], and comparative lipidomic profiling of human liver tissues was not done so far [61]. Moreover, HCV genotype of half of the patients in our study was unknown. Despite these limitations this is to our knowledge the first comprehensive analysis of the non-tumor and tumor liver lipidome in HBV and HCV mono-infected and co-infected patients.

To summarize, the associations of HBV and HCV infections with lipid metabolism have long been noticed. Aberrant lipid metabolism is a hallmark of chronic liver diseases, and furthermore, contributes to carcinogenesis. Lipids are attractive targets for drug development. Present analysis demonstrated that primary liver cancer associated changes in lipid composition vary with viral disease etiology. Moreover, PU phospholipids were low in HCV infected non-tumorous liver tissues when compared to HBV infected patients. Viral disease etiology impacts on the liver lipidome and this may be relevant for disease pathophysiology and therapy.

## 4. Materials and Methods

### 4.1. Patients

HCC tissues and para-tumorous tissues of patients infected with HBV, HCV or both viruses were obtained from resections. The study was conducted according to the guidelines of the Declaration of Helsinki. Experimental procedures accorded to the guidelines of the charitable state controlled foundation Human Tissue and Cell Research (HTCR). The study was approved by the local ethical committee of the University Hospital of Regensburg (Ethic code: 15-101-0052, approval date: 26 March 2015). Written informed consent was obtained from all participants.

The characteristics of the participants were summarized in Table 3. Tissues of patients with viral infections were used in a previous study to measure chemerin and CMKLR1 in the liver tissues [62].

### 4.2. Lipidomics

#### 4.2.1. Internal Standards

Lipid species were annotated according to the proposal for shorthand notation of MS-derived lipid structures [63]. The following lipid species were added as internal standards: CE 17:0, CE 22:0, Cer 18:1;O2/14:0, Cer 18:1;O2/17:0, DG 14:0/14:0/0:0, DG 20:0/20:0/0:0, D_7_-FC, LPC 13:0/0:0, LPC 19:0/0:0, PC 14:0/14:0, PC 22:0/22:0, PE 14:0/14:0, PE 20:0/20:0, PI 17:0/17:0, PS 14:0/14:0, PS 20:0/20:0, SM 18:1;O2/12:0, TG 17:0/17:0/17:0, and TG 19:0/19:0/19:0.

#### 4.2.2. Lipid Extraction

Samples were spiked with internal standards prior to lipid extraction (solvent of standards was removed by vacuum centrifugation). An amount of 2 mg wet weight was subjected to lipid extraction according to the protocol described by Bligh and Dyer [64] with a total chloroform volume of 2 mL. An amount of 1.1 mL (for FIA-MS/MS) and 0.5 mL (for FIA-FTMS) of the separated chloroform phase was transferred into sample vials by a pipetting robot (Genesis RSP 150, Tecan, Männedorf, Switzerland) and vacuum dried. The residues were dissolved in either 1.1 mL methanol/chloroform (3:1, *v*/*v*) with 7.5 mM ammonium acetate (FIA-MS/MS) or 1.2 mL chloroform/methanol/2-propanol (1:2:4 *v*/*v*/*v*) with 7.5 mM ammonium formate (FIA-FTMS).

#### 4.2.3. Lipid Analysis

The analysis of lipids was performed by direct flow injection analysis (FIA) using either a triple quadrupole mass spectrometer (FIA-MS/MS; QQQ triple quadrupole) [65,66] or a hybrid quadrupole-Orbitrap mass spectrometer (FIA-FTMS; high mass resolution). FIA-MS/MS (QQQ) was performed in positive ion mode using the analytical setup and strategy described previously [65,66]. A fragment ion of *m*/*z* 184 was used for lysophosphatidylcholines (LPC) [67]. The following neutral losses were applied: Phosphatidylethanolamine (PE) 141, phosphatidylserine (PS) 185, and phosphatidylinositol (PI) 277 [68]. Sphingosine based ceramides were analyzed using a fragment ion of *m*/*z* 264 [69].

A detailed description of the FIA-FTMS method was published recently [70,71]. Triacylglycerols (TG), diacylglycerols (DG) and cholesteryl esters (CE) were recorded in positive ion mode as [M + NH4]^+^ in *m*/*z* range 500–1000 and a target resolution of 140,000 (at 200 *m*/*z*). CE species were corrected for their species-specific response [72]. Phosphatidylcholines (PC) and sphingomyelins (SM) were analyzed as [M + HCOO]^−^ in negative ion mode in *m*/*z* range 520–960 at the same resolution setting. Multiplexed acquisition (MSX) was applied for the [M + NH4]^+^ of free cholesterol (FC) and the respective internal standard (D_7_-FC) [72]. Data processing details were described in Höring et al. [70] using the ALEX software [73] for peak assignment.

### 4.3. Immunoblot Analysis

Immunoblot was performed as described in detail [74]. The p53 antibody was from Santa Cruz (Dallas, TX, USA). Phospho-p53 antibodies were ordered from Cell Signaling (Frankfurt am Main, Germany). Quantification of immunoblots was done as described [75].

### 4.4. Statistical Analysis

Data are summarized with boxplots, which display the median value, the range of the values, the lower and upper quartiles. Small circles are outliers greater than 1.5 times the interquartile range and stars are outliers greater than 3.0 times the interquartile range. Data are also shown as bars, and median values are presented. Statistical analysis was done by one-way ANOVA with post-hoc Bonferroni, Kruskal-Wallis-Test, Mann-Whitney U Test or Spearman correlation (SPSS Statistics 25.0 program, IBM, Leibniz Rechenzentrum, München, Germany) and Students’ *t*-test (MS Excel, Microsoft; Redmond, WA, USA).

## Figures and Tables

**Table 1:** Phospholipids in non-tumor (NT) and tumor tissues (TT) of the 10 HBV, 11 HCV and 3 double-infected patients. Levels of saturated PE and PS were below 0.001 nmol/mg and are not included. Lipid concentrations (nmol/mg) are given as median values and range. Phosphatidylcholine, PC; phosphatidylethanolamine, PE; phosphatidylinositol, PI; phosphatidylserine, PS; polyunsaturated, PU; saturated, sat. * *p* < 0.05 and ** *p* < 0.01 and *** *p* < 0.001 compared to the respective NT using a paired Students’ *t*-test. Significantly different lipid levels in NT and TT are in bold.

	HBV		HCV		HBV/HCV	
	NT	TT	NT	TT	NT	TT
Sat. PC	**0.23** **(0.18–0.32)**	0.49 * **(0.15–1.00)**	**0.28** **(0.18–0.55)**	0.43 * **(0.11–1.08)**	0.29 (0.26–0.34)	0.32 (0.12–0.70)
PU-PC	**10.2** **(6.5–14.7)**	5.9 *** **(3.2–8.6)**	8.4 (3.3–10.2)	7.6 (0.6–16.8)	9.6 (7.6–11.1)	7.5 (1.7–10.6)
PU-PE	**7.2** **(3.8–9.9)**	4.7 ** **(0.1–8.0)**	5.0 (1.7–6.9)	6.4 (0.1–10.6)	5.4 (5.1–7.2)	6.8 (1.1–7.1)
PC/PE	1.7 (1.6–2.2)	2.1 (1.5–74.6)	2.1 (1.7–3.0)	1.9 (1.7–12.4)	2.0 (1.8–2.1)	2.1 (1.9–2.1)
Sat. PI	0.02 (0.01–0.03)	0.01 (0.00–0.03)	0.02 (0.01–0.05)	0.03 (0.00–0.07)	0.02 (0.02–0.02)	0.02 (0.01–0.10)
PU-PI	3.9 (2.3–5.6)	4.1 (0.1–6.4)	**3.0** **(1.4–4.8)**	4.7 * **(0.1–9.6)**	3.8 (3.5–4.2)	5.6 (0.8–7.8)
PU-PS	**1.8** **(1.1–2.2)**	1.2 *** **(0.1–1.9)**	1.4 (0.7–1.8)	1.5 (0.1–2.2)	1.7 (1.4–1.8)	1.2 (0.6–1.6)
Sat. LPC	0.07 (0.04–0.10)	0.08 (0.04–5.81)	0.06 (0.02–0.26)	0.06 (0.03–0.10)	0.08 (0.07–0.18)	0.04 (0.02–0.05)
PU-LPC	0.40 (0.23–0.86)	0.38 (0.21–0.81)	0.17 (0.06–0.31)	0.14 (0.07–0.29)	0.06 (0.05–0.13)	0.03 (0.01–0.05)

**Table 2:** Correlation of p53S9, S15, S20 and S392 with p53, free cholesterol, grading and T-stage in the HCC tissues of all patients. * *p* < 0.05, ** *p* < 0.01, *** *p* < 0.001.

	p53S9	p53S15	P53S20	p53S392
p53	−0.018	−0.052	0.327	0.347
p53S9		0.916 ***	0.685 *	0.685 *
p53S15	0.916 ***		0.755 **	0.598 **
p53S20	0.685 *	0.755 **		0.797 **
p53S392	0.587 *	0.598 **	0.797 **	
Free cholesterol	0.073	0.128	0.027	−0.129
Grading	−0.194	−0.033	−0.065	0.222
T-stage	−0.130	−0.072	−0.145	0.063

**Table 3:** Study group included patients infected with HBV, HCV or both viruses. GGT of 9 HBV infected patients was documented. This was indicated by an upper-case number. *p* < 0.05 between the two groups which were both labeled with *. Body mass index, BMI; alanine aminotransferase, ALT; aspartate aminotransferase, AST; gamma-glutamyl transferase, GGT, not defined, nd.

	HBV	HCV	HBV/HCV
**Number**	10	11	3
**Sex (male/female)**	8/2	8/3	2/1
**Type 2 diabetes**	2	3	0
**Age (years)**	60 (35–78)	54 (48–71)	61 (54–76)
**BMI (kg/m²)**	24.5 (18.7–29.4)	25.2 (18.8–28.7)	26.2 (22.4 –30.0)
**AST (U/L)**	40 (19–103) *	75 (39–151)	95 (78–200) *
**ALT (U/L)**	40 (24–123) *	60 (27–145)	134 (66–167) *
**Bilirubin (mg/dL)**	0.7 (0.3–1.6)	0.7 (0.4–3.7)	0.5 (0.5–0.8)
**GGT (U/L)**	97 (34–200)^9,^*	271 (55–582) *	102 (84–240)
**Steatosis Grade** **0/1/2/3/nd**	7/1/2/0	6/2/1/1/1	2/0/0/1/0
**Fibrosis Stage** **0/1/2/3/4**	2/2/0/3/3 *	0/0/0/3/8 *	0/0/1/1/1
**Grading G1/G2/nd**	1/4/5	0/8/3	0/1/2
**Staging T1/T2/T3/nd**	2/3/1/4	3/3/2/3	1/0/1/1

## Data Availability

Data supporting reported results can be obtained from the corresponding author on request.

## Supplementary Materials

The following are available online at https://www.mdpi.com/article/10.3390/ijms22105297/s1, Figure S1: title: Cholesteryl ester (CE), diacylglycerol (DG), triacylglycerol (TG) and free cholesterol (FC) in HCV and HBV/HCV patients stratified for HCV genotype.
