# Supplementary material for: Liver Lipids of Patients with Hepatitis B and C and Associated Hepatocellular Carcinoma
Source: Int J Mol Sci. 2021 May 18;22(10):5297. doi: 10.3390/ijms22105297 (PMC8157577; doi:10.3390/ijms22105297)
Supplement: Supplementary file 1 [file ijms-22-05297-s001.zip › ijms-1149552-supplementary.pdf]

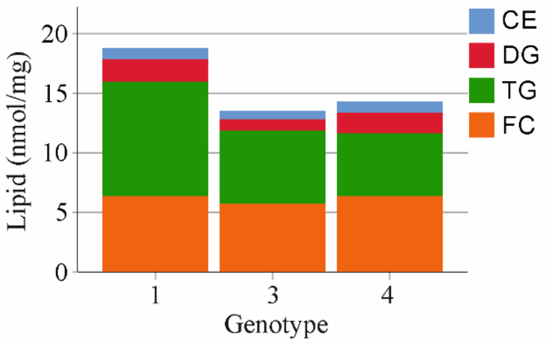

Supplementary Figure S1. Cholesteryl ester (CE), diacylglycerol (DG), triacylglycerol (TG) and free cholesterol (FC) in HCV and HBV/HCV patients stratified for HCV genotype. 4 patients had genotype 1, 2 patients had genotype 3 and 1 patient genotype 4.
